# Society Meetings

**Published:** 1913-08

**Authors:** 


### SOCIETY MEETINGS.

*Brief reports and announcements of meetings of societies of Western New York and of those of general scope are requested from secretaries. Copy should be on hand by the fifteenth of each month. Full transactions will be published at cost of composition.*

First National Congress (French) for the Protection of Early Life. May 9-11, 1913. (Abstracted from the Jour. de Med. de Bordeaux.)

Paul Morel calls attention to the considerable difference in the relation of birth and death rates in different parts of France. The nine chief cities of the southwest, including Bordeaux and Toulouse, had a total of 9,203 births and 12,531 deaths last year, while the six cities of the north had a total of 12,205 births and 10,809 deaths. The summer of 1911 was terribly hot and caused an unusually high infantile mortality all over Europe. More than 100,000 infants died, and the author estimates that 90,000 should have been saved that year and 50,000 in normal years.

Moussous and Leuret presented a paper on weaning, excellent, but containing nothing particularly new.

Several reports were made regarding amelioration of the conditions under which children are raised, with suggestions as to medical inspection, popular instruction, reporting cases, etc. About 190,000 infants are turned over to wet nurses every year, and, while the Roussel law has exercised considerable control over the nurses, there is no provision and no authority for the direct supervision of the nursling. The ideal method would be the general institution of creches like that at Porchefontaine, but this was not regarded as practicable.

Felhoen discussed the nourishment of infants whose mothers work in factories. Those infants which are entrusted to neighbors or relatives are rarely wet nursed and the mortality is about 86 per cent. Creches are expensive relatively to their use and benefit but are inevitable. The best solution is the establishment of rest rooms in factories where mothers can nurse their infants during working hours, no woman being allowed to return to work till a month after the birth. Mercier described an industrial creche of this kind at the shop of the widow of Albert Chabrat. At an average cost of 2 cents a week per employee, a daily indemnity is paid to each mother of 40 cents a day, for a month before and after labor, with an additional $2 if she nurses the child. An infant's outfit is also furnished and a cradle is loaned for a year. The Bordeaux League against Infant Mortality also maintains a creche, where infants are cared for, their linen washed and toilet necessities are supplied. Soup is also furnished free to the mothers to induce them to report regularly to nurse the children, at 10 and 4 o'clock.

Senator Paul Strauss stated that the mortality of legitimate infants was 10 per cent., that of illegitimate 22.2 per cent. He advocated public assistance to the girl-mothers and legalized inquiry as to paternity. The birth of feeble and premature infants should be prevented by prophylaxis against alcoholism, tuberculosis and syphilis. The mortality for nurslings is 131 per mille, of artificially reared infants 182 per mille. A third of the mortality of the first year occurs in the first month. The value of a campaign against the influence of ignorance and poverty is shown by the decline of the mortality for the first year of life from 17.8 per mille for the period of 1872-5 to 13.01 for the period 1906-9. But the possibility of still better results is shown by the Norwegian statistics for the latter period, 7.01 per mille. (Note—There appears to be some discrepancy between the statistics given. We have reduced the last figures from deaths per 100,000. It would seem that percentages should be substituted for per mille figures.)

Various pledges were adopted as to support and advocacy of legislation. We do not reproduce them, as they are not directly applicable to American conditions and the general principles involved are well established.

---

The Lake Keuka Medical Association held its annual meeting July 17 and 18. The following officers were elected: President, Dr. Floyd S. Crego, Buffalo; vice-President, Homer J. Knowlton, Geneva; Secretary-Treasurer, Dr. E. Carlton Foster, Penn Yan; Recording Secretary, Dr. W. W. Smith, Avoca.

La Societe D'Hygiene de L'Enfance was organized in 1887. It holds monthly meetings, with intervening meetings of the council, has physicians and philanthropists of both sexes as members, publishes a monthly bulletin, and deals broadly with the various problems of child welfare. The president is Dr. P. Guillaumet. The administrative and publication office is at 10 rue St. Antoine, Paris.

A series of four medals and honorable mentions will be awarded at the next annual meeting to essayists engaging in a competition closed with the last day of 1913. The subject for discussion is "The Place Which Ideas of Puericulture and Hygiene of Childhood Should Occupy in Modern Education." Essays may be presented in French, English, German, Italian or Spanish, designated by a motto or device, duplicated on a sealed envelope containing the name and address of the competitor.

"The Question of Depopulation," by Dr. Garnier, Sec.-Gen. of the Society. In the 17th Century the population of France was about 20,000,000. At the time of the Franco-Prussian War of 1870-71, it was 40,000,000, of which 1,500,000 were ceded with Alsace-Lorraine. Since then the population has remained nearly stationary, while that of Germany has increased one-third. The author suggests that the political solidarity of Germany and her policy of militarism accounts for the rapid increase in population and quotes Napoleon's answer to Mme. de Stael's question. "What is the quality that your Majesty most appreciates in woman?" "That of furnishing me soldiers." Thus, while war was formerly regarded as a terrible factor in diminishing population or even as an ultimately favorable remedy of super-population, it is now the end for which the increase of population, stimulated in various ways, is the means. The author also emphasizes the fact that the death rate is more important than the birth rate in an increase of population, since, even if not phenomenally high, the former indicates the prevalence of diseases which prevent long life of mothers, produce sterility, as syphilis, gonorrhoea, etc., produce poverty and hence discourage child bearing, and surround the child actually born with unfavorable factors as to careful rearing, even if not directly involving infection and hereditary transmission. Alcoholism, tuberculosis and syphilis are regarded as the chief diseases concerned in relative depopulation.

The Hygiene of Nurslings in the country. Dr. Rousseau pleads especially for the "assisted" children which are sent to the country, usually to well disposed and charitable persons. He advocates some system of co-operation between the monthly inspector for the Society and the local physician and some charitable lady so that child and nurse may not be absent at the time of the inspection, and to provide for prompt attention to minor ailments at their incipience; also for instruction in the general principles of hygiene of the persons caring for these assisted children.

The June Bulletin also reviews various articles dealing with the action of the sun's rays. While advocating the treatment of tuberculosis in high altitudes or along the ocean at places where there is abundance of sunshine, attention is called to the possible danger of sunburn and experiments with color filters are cited to show that this action is due to ultra-violet rays. The opposing influence of lightning and aurora borealis in increasing and decreasing the amount of ozone in the atmosphere is mentioned and various problems of physics of light as applied to physiologic effects are suggested.

Note—The Bulletin of the Society is received regularly at the office of the BUFFALO MEDICAL JOURNAL, and we should be glad to have it used by some local organization interested in child welfare.

---

MONROE COUNTY BRANCH OF N. Y. STATE SANITARY OFFICERS' ASSOCIATION. This body combined business and pleasure July 24, the members going by automobiles to Straight Lake, discussing the new Public Health Law and a good dinner, and returning to their homes by moonlight.

